# Impedance-based detection of cochlear implant array migration: case report in a child with Aymé-Gripp syndrome

**DOI:** 10.1007/s00405-025-09397-7

**Published:** 2025-04-29

**Authors:** Stephan Schraivogel, Sabrina Regele, Nora M. Weiss, Markus Wirth, Barbara Wollenberg, Marco Caversaccio, Wilhelm Wimmer

**Affiliations:** 1 Department of ENT, Head and Neck Surgery, Inselspital, Bern University Hospital, Bern, Switzerland; 2 ARTORG Center for Biomedical Engineering Research, University of Bern, Bern, Switzerland; 3 Department of Otorhinolaryngology, Klinikum Rechts Der Isar, Technical University of Munich, Trogerstraße 32, 81675 Munich, Germany

**Keywords:** Pediatric cochlear implantation, Revision surgery, Radiation exposure, Objective monitoring

## Abstract

**Purpose:**

Detection of complications during rehabilitation and postoperative follow-up after cochlear implantation is essential, especially in children and cognitively impaired patients. Electrode array migration can affect outcomes and must be detected early. Traditional radiographic methods, although effective, are costly and expose patients to radiation. This case report discusses the use of a previously published impedance-based model for cochlear implant array localization in a child with Aymé-Gripp syndrome.

**Methods:**

Impedance telemetry data and X-ray images were collected at the time of initial surgery and before and after the required revision surgery. The impedance-based model was used to estimate the insertion depth of the most basal cochlear implant electrode within the cochlea. The resulting estimates were compared with the electrode positions from radiographs to assess the accuracy and applicability of the model.

**Results:**

20 months after implantation, the patient suddenly stopped tolerating the CI audio processor. Retrospectively, the impedance-based model revealed substantial electrode migration, which was confirmed by postoperative radiography.

**Conclusion:**

The proposed model, which uses routine impedance telemetry data without radiation exposure, offers a cost-effective alternative to radiography. Early detection and intervention, particularly in complex cases, improves outcomes and reduces costs, highlighting the importance of objective monitoring.

## Introduction

Cochlear implants (CIs) are neuroprostheses for treating sensorineural hearing loss through electrical stimulation of the cochlear nerve. Especially in children and patients with limited ability to cooperate (e.g., syndromes), objective procedures are required to detect potential complications during the rehabilitation phase and in the later follow-up [1].

A full and secure placement of the CI array is essential for obtaining successful clinical results. During follow-up, the migration of CI contacts is a complication that is likely underreported. It can lead to reduced speech perception and requires timely revision surgery in severe cases [2]. Postoperative verification of correct surgical electrode positioning is performed using radiography, e. g., computed tomography (CT) or X-ray projections. These examinations, however, incur costs, require expert personnel, and expose the patients to radiation. Particularly, pediatric patients are reported to be at increased risk of developing leukemia and brain tumors after CT scans [3].

As an alternative, electrical impedance-based models have recently been reported to allow estimation of electrode positions from radiation-free telemetry recordings, routinely performed in CI users [4]. Here, we applied the best-performing estimation model, i.e., the decision-tree based model “Extra Trees”, as detailed in a previous study [4]. As input, the algorithm requires voltage matrices recorded using standard CI impedance telemetry protocols and cochlear dimensions measured from pre-operative radiography. As output, it estimates the linear insertion depth of the most basal electrode from the round window (in mm). Extracochlear electrodes are indicated by a negative linear insertion depth. This approach enables objective monitoring of electrode positions during follow-up visits without exposing the patient to radiation.

In this report, we present radiation-free detection of severe CI electrode migration in a child with Aymé-Gripp syndrome, a multisystemic disorder that can lead to sensorineural hearing loss.

## Case report

Publication of this case report was approved by our local institutional review board (ID 2023–367-S-SR). Written consent was obtained to present the case. We report the case of a 3.5-year-old female with evidence of a mutation of the MAF gene (16q23.2) and confirmed Aymé-Gripp syndrome [5]. The patient presented congenitally with bilateral sensorineural hearing loss, cataracts, muscular hypotonia, and neurodevelopmental abnormalities. As part of the screening procedure for CI candidacy, no reproducible potentials could be measured using evoked response audiometry. Following unremarkable CT and magnetic resonance imaging examinations, the left ear was implanted with a CI at the age of 12 months. Bilateral CI provision in the right ear followed about two months later. This report concerns the CI implanted in the left ear (FLEX^28^ MED-EL GmbH).

**Fig. 1:**
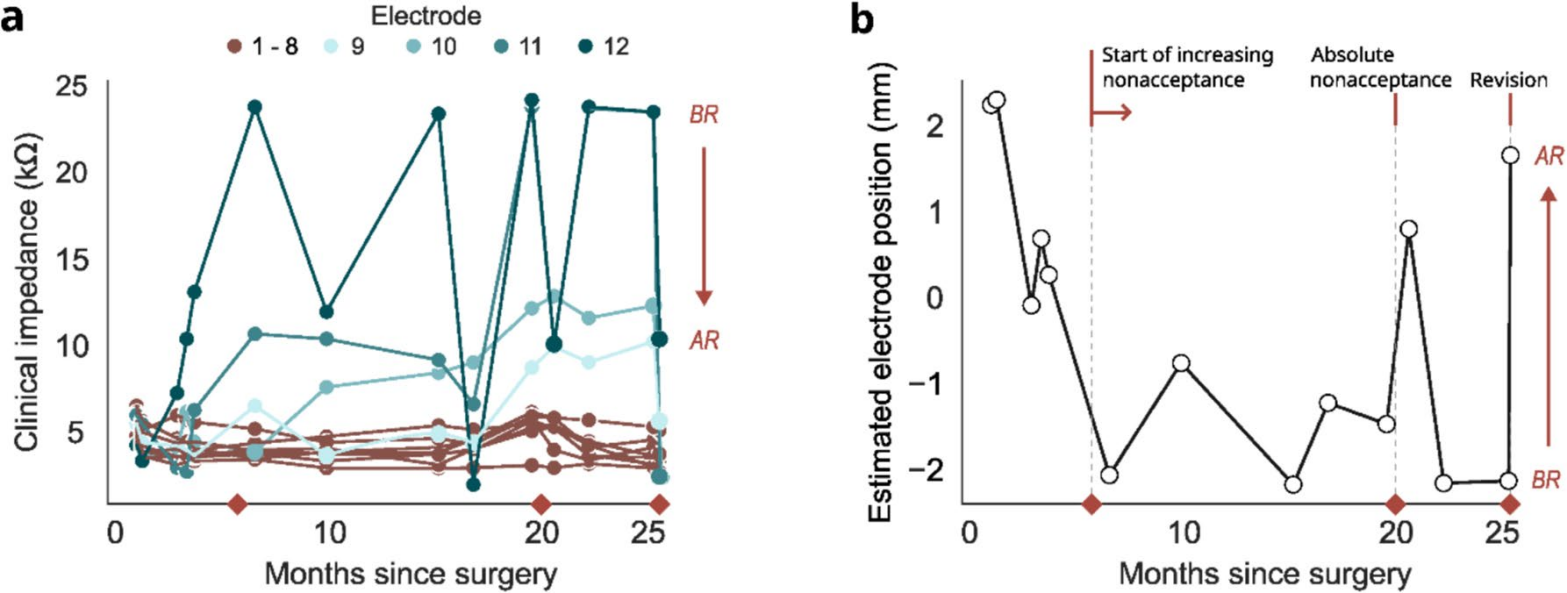
**a** Progression of clinical impedances (in kΩ,) starting from CI-surgery until revision and **b** impedance-based position monitoring of the most basal electrode contact (in mm), with positive values indicating an intracochlear and negative values an extracochlear electrode position. Important time points of clinical events are indicated with red diamonds on the x-axis. Impedance measurements were taken just before and immediately after the revision surgery, with a 90-min interval in between. BR: before revision, AR: after revision.

After activation, the patient responded well and exhibited hearing responses suitable for her developmental stage. However, 20 months after implantation, the patient suddenly stopped tolerating the CI audio processor on the left side without recognizable external influences. Fluctuations in electrode impedances over several months suggested possible electrode migration, however, did not provide a clear conclusion (Fig. 1a). Successive deactivation of electrode contacts and the adaptation of the frequency maps did not lead to an improvement.

**Fig. 2:**
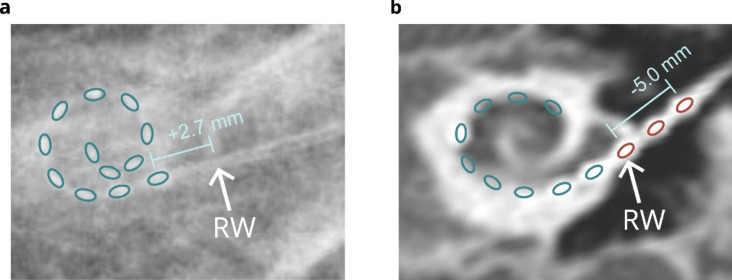
Comparison of postoperative radiographs showing the insertion depth of CI electrodes implanted in the left ear. Linear insertion depth into the cochlea was measured along the electrode array starting from the round window (RW), as indicated by the white arrow. **a** Plain radiography one day after the surgery. All electrodes are fully inserted into the cochlea, with a linear insertion depth of the most basal electrode of 2.7 mm. **b** CT scan approx. 22 months after the surgery and before the revision surgery. Three extracochlear electrodes (shown in red) indicate a substantial emigration, with a linear insertion depth of the most basal electrode of − 5.0 mm

The retrospective evaluation using the impedance-based electrode insertion depth estimation model [4] revealed substantial electrode migration, resulting in at least 2 extracochlear contacts (Fig. 1b). The emigration of electrodes was confirmed by comparing postoperative images (Fig. 2). X-ray radiographs taken immediately on the days after cochlear implantation (i.e., 1 day and 1.5 months after surgery in the left ear, respectively) showed an initially fully inserted electrode array. The CT scan, conducted 22 months post-surgery, confirmed the presence of three extracochlear electrodes. This indicated a displacement of the most basal electrode by 7*.*7 mm out of the cochlea (Fig. 2b).

**Fig. 3:**
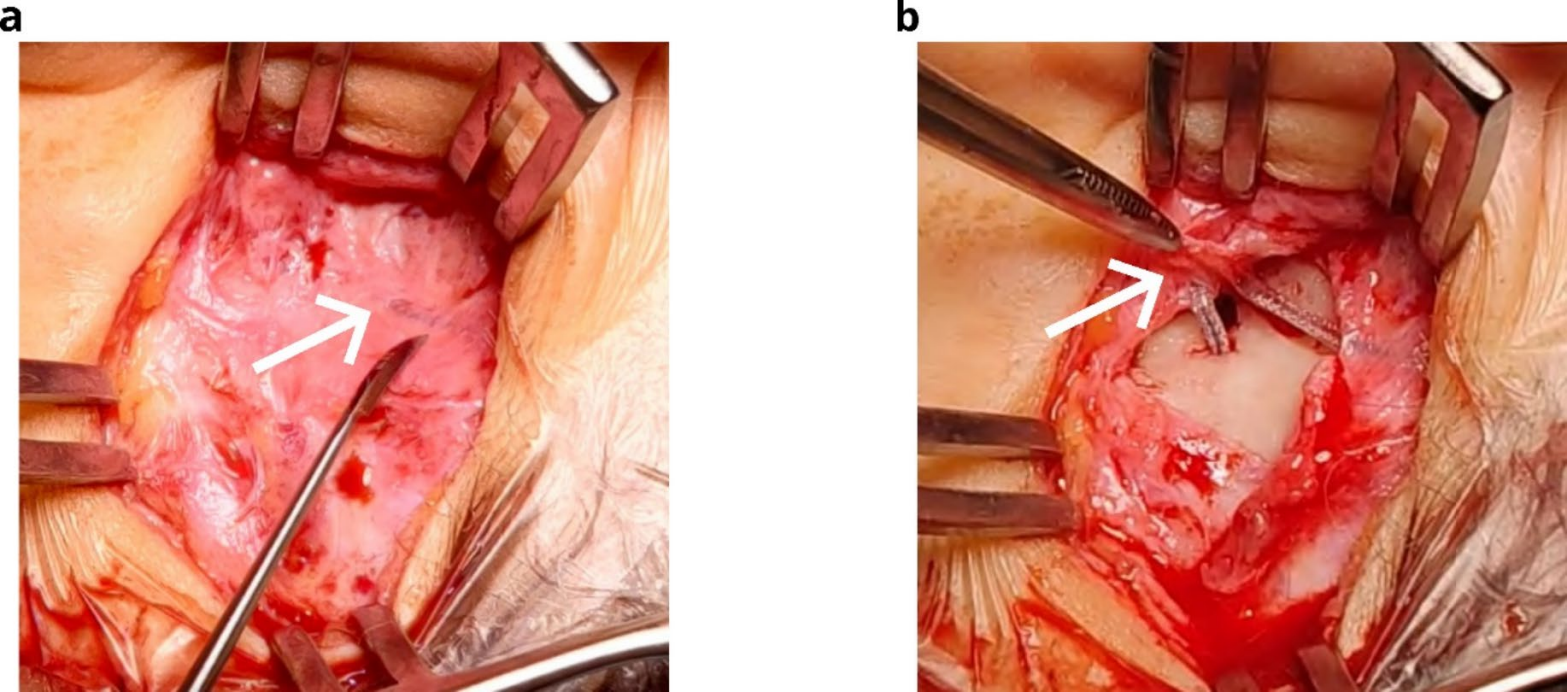
Revision surgery with the electrode array covered by the periosteum, as indicated by the white arrows. **a** Visible electrode array underneath the periosteum after the opening of the scar from the previous operation. **b** Successive opening of the periosteum while protecting the electrode array

Consequently, a revision surgery was performed 25 months after implantation. The impedances of the CI were recorded before and after the surgery to confirm the position correction. Examination of the site revealed that the array had grown into the periosteum (Fig. 3). Successive opening of the mastoidectomy showed partial re-ossification. For the revision, the posterior tympanotomy was carefully extended, and the electrode array was slowly reinserted for full insertion. The round window was sealed and the implant was fixed to the bone with periosteal sutures. Impedances at the most basal electrodes returned to normal levels (Fig. 1a) and neural responses detected on all electrodes. Based on the impedance measurement after reinsertion, the estimation model confirmed the full insertion of the electrode array (Fig. 1b). After the revision, the patient showed improved acceptance of the audio processor.

## Discussion

In this case report, we demonstrate the relevance of objective methods for detecting electrode migration in follow-up. The presented case of Aymé-Gripp syndrome was characterized by a challenging follow-up, because of the limited ability of the patient to communicate the reason for her dissatisfaction. The impedance-based position estimation model indicated migration of the electrode contacts based on the impedance recording 6 months after implantation (i.e., migration presumably occurred within this period). This coincided with the feedback of the patient’s parents, who reported a gradually increasing nonacceptance of the audio processor (Fig. 1b). According to the model’s estimates, the revision operation could have been scheduled up to 19 months earlier. Brain plasticity is high during this important learning phase, underlining the importance of early interventions.

Because the array became overgrown, the periosteum’s movement likely caused the electrode array to move (Fig. 3). This would explain the large impedance fluctuations (Fig. 1a) and the estimation of immigration by the impedance-based estimation model approximately 20 months after cochlear im- plantation (Fig. 1b).

The substantial emigration of electrode contacts (Fig. 2) was challenging for the insertion depth estimation model. It predicted a minimum linear insertion depth of the most basal electrode of around − 2*.*3 mm (Fig. 1b) compared to the ground truth of − 5*.*0 mm close to the second postoperative ground truth (Fig. 2b). The error can be explained because the insertion depth estimation model was trained with a maximum of one defective electrode and a maximum of two extracochlear electrodes. Nevertheless, the insertion depth estimation model showed a persistent emigration of the electrodes over time, which would have enabled earlier intervention.

While the evaluation of clinical impedances can be used to identify fluctuations, their interpretation might be ambiguous due to occurring post-surgical variations caused by device-tissue interactions, foreign body reaction, and transient physiological changes. Consequently, concluding electrode migration is nonspecific and requires extensive experience. In contrast, the algorithm integrates data from all electrodes and, most importantly, includes the far-field impedance subcomponents. This approach enables to exclude fluctuations associated with the electrode–electrolyte interface (i.e., the near-field impedance subcomponent) and provide more specific information regarding the insertion depth and potential electrode migration.

## Conclusion

In patients with limited ability to cooperate, impedance-based electrode position estimation offers a valuable tool for therapy control after cochlear implantation. To avoid radiation exposure in infant development, impedance-based position monitoring can be proposed as alternative in regular follow-up. In addition, early detection of CI conditions that require intervention increases user acceptance and saves follow-up treatment costs. However, further studies with larger pediatric cohorts need to be performed to enable wider algorithm application.
